# Guidelines and consensus for minimal residual disease-adapted therapy in multiple myeloma from the Pan-Pacific multiple myeloma working group

**DOI:** 10.46989/001c.160933

**Published:** 2026-05-04

**Authors:** Wenming Chen, Zhen Cai, Chor Sang Chim, Wee Joo Chng, Juan Du, Chengcheng Fu, Wen Gao, Ichiro Hanamura, Jian Hou, Jeffrey Shang-Yi Huang, Tadao Ishida, Chunrui Li, Aijun Liu, Vadim Ptushkin, Naoki Takezako, Raymond Siu Ming Wong, Dok Hyun Yoon

**Affiliations:** 1 Department of Hematology, Myeloma Research Center of Beijing, Beijing Chaoyang Hospital, Capital Medical University, Beijing, China; 2 Bone Marrow Transplantation Center, the First Affiliated Hospital, Zhejiang University School of Medicine, Zhejiang, China; 3 Department of Medicine, Hong Kong Sanatorium Hospital, Hong Kong, China; 4 National University Cancer Institute, Singapore; 5 Myeloma & Lymphoma Center, Shanghai Changzheng Hospital, Naval Medical University, Shanghai, China; 6 The First Affiliated Hospital of Soochow University, Jiangsu, China; 7 Division of Hematology, Department of Internal Medicine, Aichi Medical University, Aichi, Japan; 8 Department of Hematology, Renji Hospital, School of Medicine, Shanghai Jiao Tong University, Shanghai, China; 9 National Taiwan University Hospital, Taiwan; 10 Japanese Red Cross Medical Center, Tokyo, Japan; 11 Department of Hematology, Tongji Hospital, Tongji Medical College, Huazhong University of Science and Technology, Wuhan, Hubei, China; 12 Botkin Moscow City Clinical Hospital, Moscow, Russia; 13 Nerima Hikarigaoka Hospital, Tokyo, Japan; 14 Sir Y.K. Pao Centre for Cancer & Department of Medicine and Therapeutics, Prince of Wales Hospital, The Chinese University of Hong Kong, Hong Kong, China; 15 University of Ulsan College of Medicine, Asan Medical Center, Seoul, South Korea

**Keywords:** Multiple myeloma, Minimal residual disease, MRD-adapted therapy, CD38-based therapy, Clinical practice

## Abstract

Treatment of multiple myeloma (MM) has entered the era of deep remission–oriented therapy, where minimal residual disease (MRD) has become a key indicator for treatment response and long-term prognosis, surpassing conventional complete remission criteria. However, the optimal integration of MRD into clinical decision-making remains to be established. This expert consensus offers evidence-based guidance on redefining treatment goals in the era of CD38-based therapy, incorporating MRD status into therapeutic decisions, standardizing MRD detection, and integrating new technologies to address current challenges. The consensus supports including MRD assessment in treatment evaluation for almost all MM patients and recommends a comprehensive approach that combines bone marrow, imaging, and peripheral blood–based assessments. For patients receiving CD38-based therapy, achieving and sustaining MRD negativity is strongly linked to improved outcomes. The timing and frequency of MRD testing should be adapted to treatment phases, and CD38-based therapy may be used as part of active maintenance strategies, especially in high-risk populations. Dynamic MRD monitoring is emphasized as a critical part of disease management. Both next-generation flow and next-generation sequencing are considered interchangeable for prognostic assessment, while peripheral residual disease detection is a promising direction for future disease surveillance. This consensus provides a practical framework for integrating MRD assessment into MM clinical management. Dynamic, MRD-guided treatment strategies may enable more precise risk stratification and personalized therapy, ultimately improving long-term patient outcomes.

## Introduction

Multiple myeloma (MM) is a hematologic malignancy characterized by the uncontrolled proliferation of plasma cells within the bone marrow (BM), leading to a spectrum of clinical manifestations including bone lesions, anemia, renal impairment, hypercalcemia, and immunodeficiency. In the past decade, treatment outcomes for MM have improved significantly with the introduction of novel agents such as proteasome inhibitors, immunomodulatory drugs, and monoclonal antibodies.^1–3^ The use of autologous stem cell transplantation in eligible patients and the emergence of bispecific antibodies and chimeric antigen receptor T cell therapies for relapsed disease have also contributed to these advances. As therapeutic strategies have evolved, the depth of response has become increasingly relevant in assessing patient prognosis.^2,3^ Traditional response criteria, such as complete response (CR) and stringent CR, although useful, may not fully capture the extent of residual disease burden.^2–4^ This has led to a growing emphasis on minimal residual disease (MRD) as a more sensitive and clinically meaningful marker of therapeutic efficacy.

MRD is defined as the persistence of residual malignancy at levels below the detection threshold of conventional morphologic methods, detectable by technologies that assess genetic mutations, cell surface markers, or specific DNA gene rearrangements.^4–7^ Its importance lies in its ability to provide a more refined measure of treatment response, offering prognostic insights that go beyond CR.^4,6,7^ Numerous studies have shown that achieving MRD negativity is associated with prolonged progression-free survival (PFS) and overall survival (OS), making MRD one of the most powerful predictors of long-term outcomes in MM.^8–11^ Unlike current sensitive detection methods, MRD assessment allows for the discrimination between patients who appear to be in remission but harbor residual disease and those who achieve deep responses with minimal or no detectable tumor burden.^4,6,12^

MRD detection technologies have evolved remarkably; next-generation flow (NGF) and next-generation sequencing (NGS) can detect one malignant cell among 100,000 to 1,000,000 normal cells (10^-5^ to 10^-6^ sensitivity).^6,7^ The advantages of MRD as a prognostic indicator are manifold. First, MRD negativity has been linked to favorable outcomes across different disease stages, treatment regimens, and patient subgroups.^8–11^ Second, MRD assessment offers dynamic information about disease kinetics, as repeated testing over time can reveal sustained MRD negativity or early molecular relapse, which are highly informative for predicting long-term disease control.^13,14^ Third, MRD serves as a potential surrogate endpoint in clinical trials, enabling more rapid evaluation of novel therapies and treatment strategies.^15–17^ Collectively, these benefits underscore MRD as a central biomarker in MM research and clinical practice.

Despite these advances, MM remains a biologically heterogeneous disease, with genetic alterations and complex molecular biology, which translates into highly variable clinical courses and outcomes.^4,14,18^ This heterogeneity underscores the need for tailored treatment approaches. Thus, the integration of MRD into treatment algorithms has the potential to transform the clinical management of MM. Emerging approaches such as mass spectrometry (MS) are also being explored as peripheral blood–based tools to complement BM-based MRD assessment.^19^

This consensus aims to guide MRD-adapted treatment strategies in patients with MM. In light of the growing body of supportive evidence, this document integrates results from clinical trials, real-world studies, and expert opinions. It addresses key clinical questions, such as redefining treatment goals in the era of CD38-based therapy, applying MRD status to guide therapeutic decisions, standardizing and normalizing MRD detection methods, and integrating novel technologies to overcome current limitations. By synthesizing available data and expert consensus, this paper provides evidence-based recommendations to support clinical decision-making. Ultimately, the goal is to help clinicians optimize treatment strategies, improve outcomes, and advance precision medicine for patients with MM.

## Materials and Methods

A modified Delphi consensus process was employed to develop recommendations on MRD-adapted treatment strategies in MM. The Delphi method is a well-established, consensus-driven approach that combines evidence review and expert opinion to guide clinical decision-making.^20^ This modified process involved anonymous online voting and in-person meetings by a panel of experts to synthesize current knowledge and develop practical recommendations.

Panelists were selected by an independent planning committee, separate from any funding bodies, to ensure methodological integrity. Selection criteria included active involvement in MM clinical trials, recognized contributions to the field, and broad clinical experience. This process resulted in the participation of 17 hematology and oncology experts from the Pan-Pacific region, all with extensive expertise in MM management. All panelists provided written informed consent for their involvement.

A systematic review of peer-reviewed literature was conducted to identify studies relevant to MRD-adapted therapy in patients with MM. Focusing on pivotal clinical trials, meta-analyses, real-world studies, the International Myeloma Working Group (IMWG) 2016 consensus criteria for response and MRD assessment,^21^ the European Hematology Association and European Myeloma Network (EHA-EMN) 2025 guidelines,^22^ and the National Comprehensive Cancer Network (NCCN) guidelines for MM (Version 2.2026) to ensure alignment with current clinical practice.^23^ A research steering committee synthesized the findings and formulated seven statements on online voting conducted in March 2026. These statements covered four key domains: (1) Improvement of treatment goals in the era of CD38-based therapy, (2) Utilization of MRD status to inform therapeutic decisions, (3) Standardization of MRD detection methods, and (4) Future perspectives on the integration of novel technologies. During the voting process, panelists anonymously indicated agreement or disagreement and provided suggestions for revisions.

The Grading of Recommendations Assessment, Development and Evaluation (GRADE) system was applied to assess the quality of evidence and the strength of recommendations.^24^ Evidence quality was categorized as High (A), Moderate (B), Low, or Very Low (C), depending on study design and methodological rigor. The strength of recommendations was classified as Strong (1) or Weak (2), based on the balance of potential benefits and risks. A consensus threshold of 70% agreement was predefined to indicate meaningful agreement, though not imply unanimity.^25^ Statements reaching this threshold are referred to as having “consensus” throughout the manuscript.

This manuscript integrates evidence from pivotal clinical trials, real-world studies, and expert opinion to address key clinical questions, incorporating all statements that met the predefined consensus threshold. By combining systematic evidence evaluation with a structured expert consensus methodology, this process aimed to generate practical, evidence-informed recommendations to guide the integration of MRD into the management of MM. A final joint review of the consensus document took place in March 2026, which underwent multiple rounds of revisions before finalization.

## Results

After online voting, 17 voting experts reported their agreement level with the 7 consensus statements. These statements cover redefining treatment goals in the era of CD38-based therapy, applying MRD status to guide therapeutic decisions, standardizing MRD detection methods, and integrating novel technologies to overcome current limitations. The statements are organized into four chapters outlined below and in Table 1.

**Table 1.** Summary of consensus recommendations for the integration of MRD into MM management

**Consensus Statements**	**Agreement**
Chapter 1: Upgrading treatment goals in the era of CD38-based therapy for patients with MM	
1-1. In MM, MRD testing is recommended for patients who achieve CR, particularly those receiving more effective treatment (e.g., CD38-based), and should be incorporated into the treatment evaluation process. Given the heterogeneity of MM, the limitations of MRD assessment, and operational challenges, combining MRD testing with imaging and peripheral blood assays can offer a more comprehensive evaluation of the disease (A1).	94%
1-2. Achieving and maintaining MRD negativity is strongly associated with improved prognosis in patients with MM receiving CD38-based therapy and may serve as a key treatment goal in clinical practice (A1).	100%
Chapter 2：MRD status guidance for clinical decision-making	
2-1. The timing and frequency of MRD assessment are recommended to be tailored to the treatment phase. MRD testing is recommended for patients who achieve CR following a treatment course to confirm the depth of response. During maintenance therapy, MRD-positive patients should undergo MRD testing every 6 months, while MRD-negative patients should be tested every 12 months to monitor disease status (B1).	94%
2-2. CD38-based therapy is recommended as an active maintenance approach for patients with MM, regardless of MRD status. It may provide greater relative benefit for MRD-positive or high-risk patients, potentially helping to offset their inherently poor prognosis (A1).	94%
2-3. Dynamic MRD monitoring is recommended to track disease status over time and allow timely therapeutic decisions, such as early intervention for MRD resurgence and treatment intensification for persistent MRD (B1).	100%
Chapter 3: Standardization and normalization of MRD detection	
3-1. NGF and NGS are considered interchangeable for prognostic assessment of MRD status in MM (B1).	82%
Chapter 4: Development of new detection technologies and possible solutions	
4-1. PRD detection represents a key direction for future disease monitoring (B1).	82%

MM, multiple myeloma; MRD, minimal residual disease; CR, complete response; NGF, next-generation flow; NGS, next-generation sequencing; PRD, peripheral residual disease

### Chapter 1: Upgrading treatment goals in the era of CD38-based therapy for patients with MM

If available, do you agree that MRD testing should be performed for all patients with MM?

Do you agree that MRD assessment should be incorporated into the treatment evaluation process for patients with MM?

#### Statement

In MM, MRD testing is recommended for patients who achieve CR, particularly those receiving more effective treatment (e.g., CD38-based), and should be incorporated into the treatment evaluation process. Given the heterogeneity of MM, the limitations of MRD assessment, and operational challenges, combining MRD testing with imaging and peripheral blood assays can offer a more comprehensive evaluation of the disease (A1).

#### Discussion

MRD has emerged as one of the most powerful prognostic and response assessment tools in MM.^13^ With advances in therapeutic strategies, patients are achieving deeper and more durable responses. MRD evaluation has become essential for refining response assessment in patients achieving CR.

The 2016 IMWG consensus criteria marked a milestone by incorporating MRD negativity using sensitive techniques, such as NGF and NGS, which can detect disease at a sensitivity of at least 1 in 10^5^ nucleated cells.^21^ The IMWG also emphasized that incorporating BM-based MRD assessment with multi-compartment testing and diverse technologies offers a comprehensive strategy for detecting very small amounts of disease in patients with MM. The NCCN guidelines now recommend response assessment according to IMWG criteria and MRD testing for prognostication,^23^ supporting the use of MRD assessment to guide treatment decisions.

A large body of evidence demonstrates that MRD negativity is strongly associated with improved PFS and OS.^10,16^ A pooled analysis of individual patient data (11 studies; 4,773 patients) showed that achieving MRD negativity at 9–12 months strongly correlated with improved PFS and OS across transplant-eligible newly diagnosed MM (NDMM) patients, transplant-ineligible NDMM patients, and patients with relapsed/refractory MM (RRMM). The global odds ratio (OR) for PFS ranged from 3.06 (95% confidence interval [CI], 2.09–4.03) to 16.24 (95% CI, 5.77–26.71), and for OS from 2.81 (95% CI, 1.54–4.08) to 10.34 (95% CI, 0.97–19.72).^16^ Trial-level associations were consistent for both PFS (R^2^=0.61–0.70) and OS (R^2^=0.54–0.69). Similarly, the EVIDENCE meta-analysis (8 studies; 4,907 patients) confirmed robust trial-level associations between 12-month MRD negativity and PFS (R^2^=0.53–0.91).^10^ At the individual level, global OR demonstrated the associations between 12-month MRD negativity and both PFS (4.72; 95% CI, 3.53–5.90) and OS (4.02; 95% CI, 2.57–5.46). These findings across diverse MM trials consistently show that patients achieving MRD negativity, regardless of treatment regimen, experience superior long-term outcomes compared with MRD-positive patients. In 2024, the FDA Oncologic Drugs Advisory Committee recognized the prognostic value of MRD and endorsed its use as a surrogate endpoint for the accelerated approval of new MM therapies,^26^ establishing MRD testing as a central measure of treatment response in clinical trials.

A retrospective, single-center study reported that MRD negativity (10^-5^) was not an absolute predictor, as patients with high-risk chromosomal abnormalities (HRCAs) had significantly worse survival outcomes compared with standard-risk patients (P=0.001).^27^ At a median follow-up of 14 months, relapse or death rates were 10% for standard-risk/MRD-negative, 20% for standard-risk/MRD-positive, 40% for high-risk/MRD-negative, and 45% for high-risk/MRD-positive patients (P=0.0041). These results indicate significantly worse PFS in high-risk groups, regardless of MRD status. However, interpretation may be limited by the small high-risk cohort (n=29) and the short follow-up period. Similarly, in the phase II MASTER trial, MRD-SURE was defined as patients who achieved MRD negativity after or during two consecutive phases at dynamic time points, stopped treatment, and began observation with MRD surveillance.^28^ Among the 84 patients who reached MRD-SURE, the 24-month cumulative incidence of progression was 9% for those with no HRCAs, 9% for those with one HRCA, and 47% for those with two or more HRCAs. These results show that outcomes remain unsatisfactory for patients with two or more HRCAs. Based on current limited evidence, MRD status alone does not fully mitigate the poor prognosis of high-risk patients. Future research is needed to confirm the predictive value of MRD in this group. Strategies such as increasing sensitivity, serial MRD monitoring, assessment of myeloma biology, and MRD-guided treatment may help improve survival for all patients with MM.^27^

While MRD assessment has been validated as a robust endpoint in clinical trials, its role in routine clinical practice remains less clearly defined,^29^ reflecting a translational gap between research and real-world applications. This gap arises from several challenges. First, the heterogeneity of MM means that the prognostic significance of MRD negativity may vary across biological subgroups, particularly in patients with HRCAs.^27,28^ Second, BM-based MRD testing carries inherent limitations, including patchy marrow involvement, hemodilution, and the inability to fully capture extramedullary disease, all of which may lead to underestimation or misinterpretation of residual disease. Third, resource limitations—such as access to NGF or NGS platforms, high testing costs, and the need for specialized expertise—further restrict the broad implementation of MRD assessment in daily clinical practice. Finally, frequent invasive BM aspirations may not be feasible or acceptable in long-term follow-up.^30^ These issues highlight why MRD, despite its validation in clinical trials, has not yet been universally adopted as a decision-making tool in routine practice. To address these limitations, integrating MRD assessment with imaging-based methods (e.g., positron emission tomography/computed tomography) and peripheral blood assays (e.g., MS) could ultimately provide a more comprehensive assessment of disease.^13^

#### Level of consensus on whether MRD testing should be performed in all patients with MM

94% (16) agree; 6% (1) disagree.

Total: 17 voters.

#### Level of consensus on whether MRD assessment should be incorporated into treatment evaluation in patients with MM

94% (16) agree; 6% (1) neutral.

Total: 17 voters.

Do you agree that achieving and maintaining MRD negativity is associated with better prognosis in patients with MM receiving CD38-based therapy?

#### Statement

Achieving and maintaining MRD negativity is strongly associated with improved prognosis in patients with MM receiving CD38-based therapy and may serve as a key treatment goal in clinical practice (A1).

#### Discussion

Over the past decade, anti-CD38 monoclonal antibodies have led to a new era of MM therapy.^13^ Daratumumab and isatuximab, both approved for MM, are now recommended by NCCN and EHA-EMN guidelines as preferred treatments due to their benefits in PFS and MRD negativity.^22,23^ Daratumumab has been approved for use in relapsed and frontline therapies throughout the Pan-Pacific region since 2016, initially in Singapore^31^ and followed by Japan,^32^ Russia,^33^ South Korea,^34^ and China.^35^ Isatuximab has also been approved for RRMM in the region since 2021 in Japan^36^ and subsequently in Russia, South Korea,^37^ Singapore,^38^ and China.^39^ In 2025, its indications were later expanded to include NDMM in Japan^40^ and transplant-ineligible NDMM in China.^41^ As MRD negativity is a strong predictor of a favorable prognosis in both NDMM and RRMM, many clinical trials of CD38-based therapies now use MRD as an early endpoint to assess response.^8,15^

Accumulating evidence from phase III trials clearly demonstrates that achieving MRD negativity and particularly sustaining MRD negativity leads to a better prognosis in patients with MM receiving CD38-based therapy.^42–57^ The CASSIOPEIA,^42,43^ PERSEUS,^44,45^ and GMMG-HD7^46^ studies evaluated anti-CD38-based quadruplet regimens in transplant-eligible patients and showed that those who achieved MRD negativity had longer OS and PFS. In transplant-ineligible or transplant-deferred patients, both the anti-CD38-based triplet regimen in MAIA^47,48^ and quadruplet regimens in ALCYONE,^48,49^ OCTANS,^50^ IMROZ,^51^ and CEPHEUS^52^ confirmed that sustained MRD negativity was associated with superior outcomes. The CASTOR,^53,54^ POLLUX,^54,55^ and IKEMA^56,57^ studies evaluated anti-CD38-based triplet regimens in patients with RRMM, further reinforcing this relationship. Table 2 summarizes the results of several pivotal phase III clinical trials. Notably, monoclonal antibodies targeting CD38 may interfere with the detection of plasma cells by conventional flow cytometry, potentially resulting in false-negative findings.^58^ Therefore, multi-epitope antibodies should be used instead of standard CD38 for patients receiving CD38-based therapy to ensure reliable identification of plasma cells.^59,60^ Taken together, achieving MRD negativity and maintaining it over time confers significant survival advantages for patients receiving CD38-based therapy.

**Table 2.** Phase III clinical trials integrating MRD assessment at sensitivity thresholds of 10^-5^ and 10^-6^ in MM

**Study**	**Treatment**	**MRD Assessment Timepoints**	**Rates of MRD Negativity (10^-5^)**	**Rates of Sustained MRD Negativity (10^-5^)**	**Rates of MRD-CR (10^-5^)**	**Rates of MRD-CR (10^-6^)**	**Median PFS (months)**	**Median OS (months)**
CASSIOPEIA (transplant-eligible)^42,43^	Part 1 (induction to consolidation): Dara-VTd Part 2 (maintenance) Dara vs observation	Prespecified intervals during maintenance or observation (≥VGPR) Prespecified intervals during follow-up in non-progressive, MRD-negative patients	77.3% vs 70.7% (P=0.0417)	≥36 months: 43.7% vs 32.3% (P=0.0088)	≥36 months: 43.2% vs 31.0% (P=0.0047)	≥36 months: 29.3% vs 22.3% (P=0.0807)	NR vs 72.1 HR, 0.76 (P=0.048)	NR vs NR
	Part 1 (induction to consolidation): VTd Part 2 (maintenance) Dara vs observation		70.9% vs 51.2% (P<0.0001)	≥36 months: 31.9% vs 12.1% (P<0.0001)	≥36 months: 31.5% vs 11.6 (P<0.0001)	≥36 months: 19.7% vs 6.5 (P<0.0001)	NR vs 32.7 HR, 0.34 (P<0.0001)	NR vs NR
PERSEUS (transplant-eligible)^44,45^	Dara-VRd vs VRd	After consolidation (≥VGPR) Suspected ≥CR	75.2% vs 47.5% (P<0.001)	≥24 Months: 55.8% vs 22.6%	75.2% vs 47.5% (P<0.001)	65.1% vs 32.2%	NR vs NR HR, 0.42 (P<0.001)	NR vs NR
GMMG-HD7 (transplant-eligible)^46^	Isa-VRd vs VRd	After induction After transplant	After transplant: 66.2% vs 47.7% (P<0.0001)	Not reported	After transplant: 38.1% vs 25.8% (P=0.001)	Not reported	NR vs NR HR, 0.70 (P=0.0184)	NR vs NR
MAIA (transplant-ineligible)^47,48^	Dara-Rd vs Rd	Baseline Suspected ≥CR Prespecified intervals (≥CR)	32.1% vs 11.1% (P<0.0001)	≥18 months: 16.8% vs 3.3% (P<0.0001)	58.2% vs 34.0% (P=0.0001) ≥12 months: 22.0% vs 9.0% (P=0.0053)	Not reported	61.9 vs 34.4 HR, 0.55 (P<0.0001)	90.3 vs 64.1 HR, 0.67 (P<0.0001)
ALCYONE (transplant-ineligible)^48,49^	Dara-VMP vs VMP	≥CR Prespecified intervals after the first treatment dose	28% vs 7% (P<0.0001)	≥12 months: 14% vs 3% (P<0.0001)	58.8% vs 27.8% (P<0.0001) ≥12 months: 30.6% vs 11.1% (P=0.0006)	Not reported	66.7 vs 42.4 HR, 0.56 (P<0.0001)	83.0 vs 53.6 HR, 0.65 (P<0.0001)
OCTANS (transplant-ineligible)^50^	Dara-VMP vs VMP	≥CR Prespecified intervals after the first treatment dose (≥CR)	40.4% vs 10.8% (P<0.0001)	≥18 months: 15.1% vs 1.4% (P=0.0008)	Not reported	Not reported	38.7 vs 19.2 HR, 0.35 (P<0.0001)	NR vs NR HR, 0.60 (P=0.0600)
IMROZ (transplant-ineligible)^51^	Isa-VRd vs VRd	Baseline After induction (≥VGPR) During continuous treatment (≥VGPR)	58.1% vs 43.6%	≥12 months: 46.8% vs 24.3%	55.5% vs 40.9% (P=0.003)	40.0% vs 22.7%	NR vs 54.3 HR, 0.60 (P<0.001)	NR vs NR
CEPHEUS (transplant-ineligible or transplant-deferred)^52^	Dara-VRd vs VRd	Baseline Suspected CR Prespecified intervals after the first treatment dose Annually (≥CR)	60.9% vs 39.4% (P<0.0001)	≥12 months: 48.7% vs 26.3% (P<0.0001)	60.9% vs 39.4% (P<0.0001)	46.2% vs 27.3% (P=0.0001)	NR vs 52.6 HR, 0.57 (P=0.0005)	NR vs NR
CASTOR (RRMM)^53,54^	Dara-Vd vs Vd	Suspected CR Prespecified intervals after the first treatment dose Annually (≥CR)	15.1% vs 1.6% (P<0.0001)	≥12 months: 6.8% vs 0.0% (P<0.0001)	52.8% vs 17.4% (P=0.0035) ≥12 months: 23.6% vs 0.0% (P=0.0098)	Not reported	13.2 vs 9.2 HR not reported	49.6 vs 38.5 HR, 0.74 (P=0.0075)
POLLUX (RRMM)^54,55^	Dara-Rd vs Rd	Suspected CR Prespecified intervals (≥CR)	33.2% vs 6.7% (P<0.0001)	≥12 months: 16.1% vs 1.4% (P<0.0001)	57.4% vs 29.2% (P=0.0001) ≥12 months: 28.4% vs 6.2% (P=0.0001)	Not reported	53.3 vs 19.6 HR, 0.42 (P<0.0001)	67.6 vs 51.8 HR, 0.73 (P=0.0044)
IKEMA (RRMM)^56,57^	Isa-Kd vs Kd	≥VGPR Confirmed best response was reached	33.5% vs 15.4%	Not reported	26.3% vs 12.2%	Not reported	35.7 vs 19.2 HR, 0.58	NR vs NR HR, 0.78
IKEMA (RRMM, East Asian subgroup)^56,57^	Isa-Kd vs Kd		44.0% vs 9.5%	Not reported	36.0% vs 4.8%	Not reported	NR vs 18.53 HR, 0.58	NR vs NR

CR: complete response; Dara: daratumumab; d: dexamethasone; HR: hazard ratio; Isa: isatuximab; K: carﬁlzomib; MRD-CR: MRD-negative complete response, which is defined as the achievement of both MRD negativity and complete response; M: melphalan; MM: multiple myeloma; MRD: minimal residual disease; NR: not reached; OS: overall survival; PFS: progression-free survival; P: prednisone; R: lenalidomide; RRMM: relapsed/refractory MM; T: thalidomide; V: bortezomib; VGPR: very good partial response.

#### Level of consensus

100% (17) agree.

Total: 17 voters.

### Chapter 2：MRD status guidance for clinical decision-making

Do you agree that the timing and frequency of MRD assessments should be tailored according to the treatment phase for patients with MM?

#### Statement

The timing and frequency of MRD assessment are recommended to be tailored to the treatment phase. MRD testing is recommended for patients who achieve CR following a treatment course to confirm the depth of response. During maintenance therapy, MRD-positive patients should undergo MRD testing every 6 months, while MRD-negative patients should be tested every 12 months to monitor disease status (B1).

#### Discussion

In MM, the optimal timing and frequency of MRD assessments have yet to be standardized, and these assessments vary significantly across clinical settings and trials.^13^ A modified Delphi study surveying MM clinicians and researchers highlighted variability in MRD testing practices.^61^ While consensus was not met on the optimal testing frequency, most respondents supported assessments within 3 months post-transplant, followed by periodic monitoring during maintenance, ranging from every 6 months to annually.

Evidence from a prospective observational study illustrates how MRD dynamics evolve over time.^62^ At a sensitivity of 10^-5^, MRD negativity rates increased from 35% post-induction to 55% at 1 year post-transplant, followed by a gradual annual decline. In line with this, a substudy of EMN02/HO95 in patients achieving CR showed that loss of MRD negativity (4x10^-5^) preceded biochemical relapse by a median of 5.5 months and clinical progression by 12.6 months, underscoring the predictive value of continuous MRD surveillance during remission.^63^ MRD testing is increasingly used to guide transplant decisions in MM, including whether patients with deep MRD negativity after induction should have stem cells collected and defer transplant until relapse.^64,65^ After transplant, MRD status can inform the need for consolidation therapy and help determine the appropriate duration of maintenance.^64–66^

**Figure 1.**
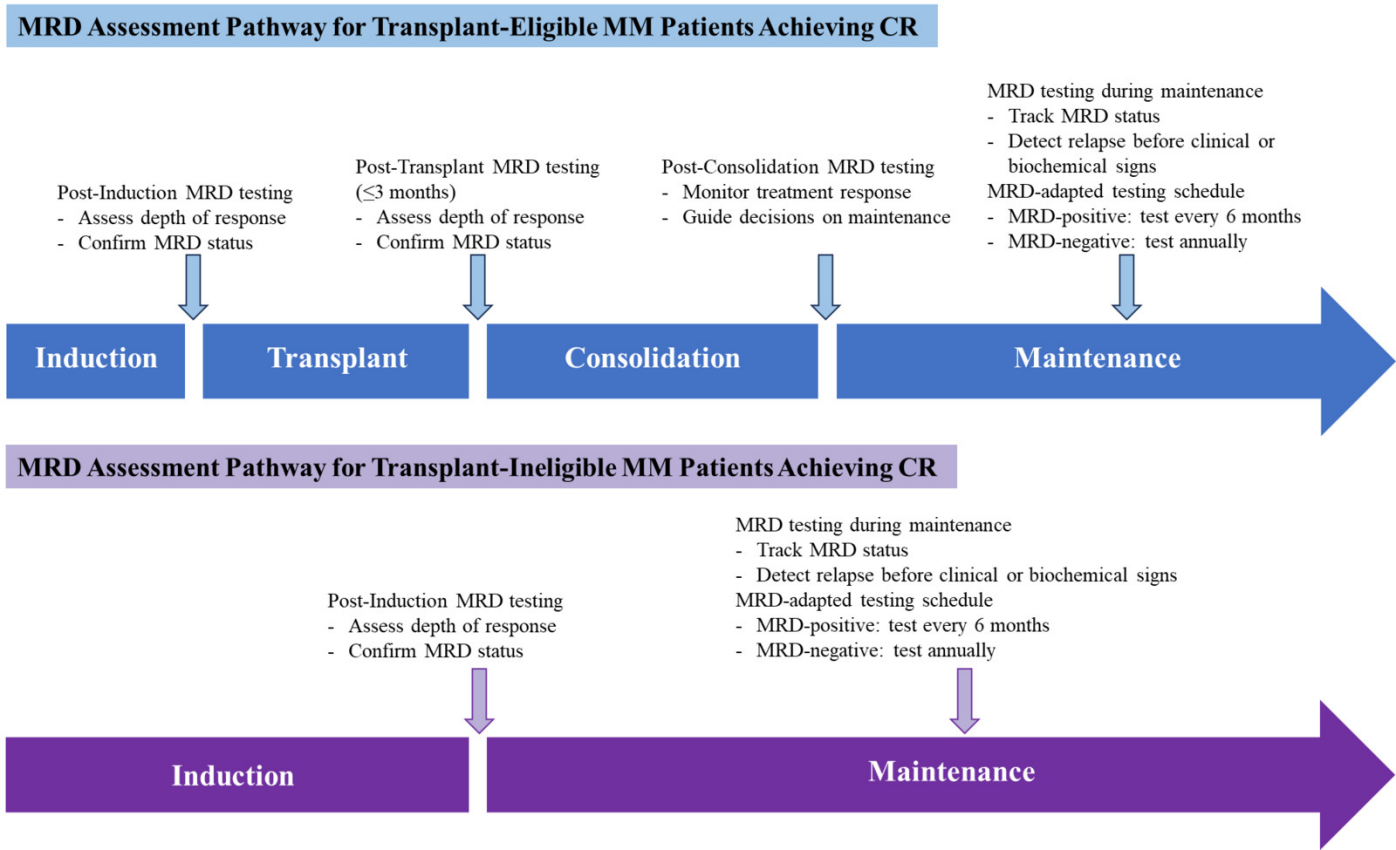
MRD-based treatment pathways for optimizing MM care CR: complete response; MM: multiple myeloma; MRD: minimal residual disease

According to the 2024 Chinese expert consensus and 2024 Chinese guideline on efficacy evaluation, the MRD assessment is typically recommended beginning when patients are considered to have achieved CR.^67,68^ Specifically, recommended time points include after induction therapy, 3 months post-transplant, or at the end of consolidation, to confirm therapeutic response. During maintenance therapy, MRD-positive patients should be tested every 6 months, while MRD-negative patients may be tested annually until relapse or progression. For non-transplant patients, MRD testing is advised at CR or after fixed-duration therapy, with the same 6-month versus annual schedule based on MRD status.^67^ While the optimal timing and frequency of MRD monitoring remain under investigation, current evidence supports testing at key milestones—post-induction, post-transplant, post-consolidation, and every 6 to 12 months during maintenance therapy.^42–44,46,48,49^ These strategies underscore the clinical utility, supporting a dynamic, risk-adapted approach to MRD assessment, which varies according to the treatment phase and MRD status, as illustrated in Figure 1.

#### Level of consensus

94% (16) agree; 6% (1) neutral.

Total: 17 voters.

Based on MRD status, do you agree that CD38-based therapy could be used for active maintenance therapy?

#### Statement

CD38-based therapy is recommended as an active maintenance approach for patients with MM, regardless of MRD status. It may provide greater relative benefit for MRD-positive or high-risk patients, potentially helping to offset their inherently poor prognosis (A1).

#### Discussion

The NCCN guidelines recommend lenalidomide as the standard-of-care (SOC) maintenance therapy after transplant in patients with MM,^23^ while the EHA-EMN guidelines now propose lenalidomide with or without daratumumab as the new SOC.^22^ The NCCN has reclassified carfilzomib-lenalidomide and daratumumab-lenalidomide as other recommended regimens, reflecting their increasing clinical relevance.^23^ In addition, two-drug maintenance is advised for high-risk patients. The NCCN has removed all maintenance options for transplant-ineligible patients and now advises continuous therapy until disease progression, with adjustments to dose and duration based on tolerance. In this context, as most patients eventually relapse, optimizing maintenance strategies to improve long-term outcomes is essential, and growing evidence supports using CD38-based regimens in this setting.^3,44,69^

The phase III AURIGA study provides direct evidence for active CD38-based maintenance therapy.^69^ It evaluated daratumumab plus lenalidomide (Dara-R) versus lenalidomide alone in CD38-based therapy naïve patients with NDMM who had undergone transplant, achieved at least a very good partial response, and remained MRD-positive (10^-5^). Dara-R significantly increased MRD-negative (10^-5^) conversion rates at a median follow-up of 40.3 months (60.6% vs. 28.7%; OR 3.92; 95% CI, 2.16–7.14; P<0.0001), MRD-negative (10^-6^) conversion rates (36.4% vs. 13.9%, OR 3.59; 95% CI, 1.78–7.23; P=0.0003), sustained MRD negativity (10^-5^) for ≥12 months (29.3% vs. 7.9%; OR 4.88; 95% CI, 2.09–11.38; P=0.0001), and improved PFS (hazard ratio [HR], 0.55; 95% CI, 0.33-0.91, P=0.0183). Subgroup analysis of patients with HRCAs further showed higher MRD (10^-5^) conversion rates (50.0% vs. 20.0%; OR 4.00; 95% CI, 0.88–18.22). Collectively, these findings directly support CD38-based active maintenance in MRD-positive patients.

In the phase III CASSIOPEIA trial, daratumumab-bortezomib-thalidomide-dexamethasone (Dara-VTd) was administered across induction and consolidation, followed by daratumumab maintenance or observation.^43^ Dara-VTd induction/consolidation plus 2 years of daratumumab maintenance improved deep MRD negativity at 10^-5^ (77.3% vs. 70.7%; P=0.0417) and sustained ≥36 months (43.7% vs. 32.3%; P=0.0088), translating into superior PFS versus observation across patient subgroups: MRD-negative patients (HR, 0.54; 95% CI, 0.38–0.78; P=0.0007), MRD-positive patients (HR, 0.48; 95% CI, 0.39–0.60; P<0.0001), standard-risk patients (HR, 0.58; 95% CI, 0.48–0.71; P<0.0001), and patients with HRCAs (HR, 0.39; 95% CI, 0.25–0.63; P<0.0001).

Similarly, the phase III PERSEUS study showed that daratumumab-bortezomib-lenalidomide-dexamethasone (Dara-VRd) given during induction and consolidation, followed by Dara-R maintenance versus VRd induction/consolidation plus lenalidomide maintenance, markedly increased MRD-CR rates (defined as MRD negativity at 10^-5^ and ≥CR; 75.2% vs. 47.5%; P<0.001), and significantly improved PFS (HR, 0.42; 95% CI, 0.30–0.59; P<0.0001).^44^

In the phase II GRIFFIN trial, Dara-VRd administered across induction and consolidation, followed by Dara-R maintenance versus VRd induction/consolidation plus lenalidomide maintenance, resulted in higher rates of deep responses and durable MRD negativity at 10^-5^ (64.4% vs. 30.1%; P<0.0001; sustained ≥12 months, 44.2% vs. 13.6%), and translated into a 55% reduction in the risk of disease progression or death (HR, 0.45; 95% CI, 0.21–0.95; P=0.0324).^70^

The strongest direct evidence for active CD38-based maintenance comes from trials that randomized daratumumab in the maintenance phase (e.g., AURIGA and the CASSIOPEIA maintenance arm).^43,69^ Other studies, although not primarily designed to evaluate CD38-based maintenance as a standalone strategy, also provide supportive evidence that CD38-based therapies can actively enhance MRD conversion and improve outcomes in the maintenance setting.^44,70^ Moreover, the phase III GMMG-HD7 study compared isatuximab-bortezomib-lenalidomide-dexamethasone (Isa-VRd) versus VRd in transplant-eligible patients; in Part 2, patients are re-randomized after transplantation to receive either isatuximab plus lenalidomide maintenance or lenalidomide alone.^46^ This ongoing study will help clarify the role of CD38-based therapy as active maintenance. Overall, CD38-based therapy has robust evidence supporting its role in active maintenance to deepen MRD responses and improve PFS, particularly in MRD-positive patients after transplantation.

#### Level of consensus

94% (16) agree; 6% (1) neutral.

Total: 17 voters.

Do you agree that dynamic MRD monitoring will provide a more precise application in future clinical practice?

#### Statement

Dynamic MRD monitoring is recommended to track disease status over time and allow timely therapeutic decisions, such as early intervention for MRD resurgence and treatment intensification for persistent MRD (B1).

#### Discussion

MRD has emerged as a critical biomarker in MM, offering insights that guide individualized treatment decisions beyond conventional response criteria.^11,12^ Serial MRD monitoring enables clinicians to evaluate the depth and durability of treatment, informing them when therapy can be safely discontinued or when intensification is warranted.^4,11^ Cumulative evidence indicates that MRD is anticipated to play a pivotal role across multiple aspects of MM management.^12,13^

First, in maintenance therapy, sustained MRD negativity may support treatment discontinuation, thereby reducing long-term toxicity and financial burden.^71,72^ The phase III GEM2014MAIN trial demonstrated that patients who were MRD-negative at 2 years and stopped maintenance had a low progression rate of 17.2% at 4 years.^73^ Moreover, the phase II MRD2STOP and MASTER studies also confirmed that discontinuing treatment is safe and feasible for patients with deep, sustained MRD negativity.^28,72^ However, more evidence is needed to clarify the role of sustained MRD negativity in guiding maintenance discontinuation.

Second, MRD status can help determine the necessity of consolidation therapy. The phase II MASTER study demonstrated that post-transplant consolidation was guided by MRD status, with treatment discontinued in patients who achieved MRD negativity (10^-5^).^28^ Patients in the study regularly received 4 cycles of daratumumab-carfilzomib-lenalidomide-dexamethasone (Dara-KRd) consolidation. An additional 4 cycles of Dara-KRd consolidation were allowed when patients failed to achieve MRD negativity. Two years after stopping therapy, less than 15% of these patients had disease progression or needed retreatment for MRD resurgence. This study shows that patients with deep MRD negativity can safely defer additional consolidation without affecting outcomes.

Third, in cases of residual disease, MRD persistence may justify treatment intensification.^4,11^ The phase III PETHEMA/GEM2012MENOS65 trial showed that MRD-positive patients after induction therapy may benefit from transplant or additional consolidation.^65^ Survival was similar between patients with MRD negativity (2x10^-6^) after induction and those who became MRD-negative after treatment intensification. The 36-month PFS rates were 88% for the group with undetectable MRD after induction and 85% for the intensification group (P=0.38), while the corresponding 36-month OS rates were 94% and 99% (P=0.17).

Finally, in MRD resurgence management, MRD monitoring may facilitate early intervention for patients with biochemical progression, potentially improving long-term disease control.^13^ Evidence from the phase II MRD2STOP study showed that patients who experienced MRD resurgence (10^-6^) during maintenance therapy discontinuation were able to regain MRD negativity after successful retreatment.^72^ The phase II MASTER study showed that among patients who transitioned to MRD-SURE, MRD resurgence before progression became more frequent over time.^28^ Five patients treated with the combination therapy of lenalidomide and daratumumab showed MRD reduction or stabilization. These findings underscore dynamic MRD monitoring as both a prognostic marker and a practical tool for adapting MM therapy, potentially guiding individualized treatment.

#### Level of consensus

100% (17) agree.

Total: 17 voters.

### Chapter 3: Standardization and normalization of MRD detection

Do you agree that NGF and NGS are largely interchangeable for prognostic assessment of MRD status in MM when performed at validated sensitivity thresholds?

#### Statement

NGF and NGS are considered interchangeable for prognostic assessment of MRD status in MM (B1).

#### Discussion

MRD assessment has become a pivotal endpoint in MM, and both NGF and NGS are widely adopted techniques to quantify residual tumor burden.^12,13,30,67,74–76^

From a methodological standpoint, NGF relies on highly standardized multiparametric flow cytometry with optimized antibody panels, typically capable of detecting malignant plasma cells at a sensitivity of 10^-5^ to 10^-6^.^30^ It has the advantage of rapid turnaround, broad accessibility, and the ability to simultaneously characterize immune reconstitution. The Chinese consensus recommends using either the 8-color two-tube or the 10-color single-tube antibody panels for MRD detection.^67^ The 10-color single-tube method reduces reagents, instrument time, and labor costs compared to the 8-color two-tube approach.^77^ Both methods show highly similar detection capabilities, with an overall concordance of 98%. By contrast, NGS interrogates immunoglobulin gene rearrangements, tracking clonotypes unique to each patient. However, NGS requires diagnostic material for sequencing and longer processing times, while NGF is feasible without baseline DNA and may be limited by sample quality or hemodilution.^30,67,76^ The key differences between NGF and NGS are summarized in Table 3.

**Table 3.** Key differences between NGF and NGS^30,67,76,78,79^

**Feature**	**NGF**	**NGS**
**Applicability**	~100%	~90%; NGS is not applicable to some patients with somatic hypermutation.
**Turnaround Time**	2–3 hours	5–7 days
**Fresh sample**	Needed (within 24-48 hours)	Not needed
**Technical Principle**	Aberrant plasma cells are detected based on immunophenotypic abnormalities in antigen expression, such as CD19, CD38, CD45, CD56, and CD138. **Note.** Monoclonal antibodies targeting CD38 may interfere with the flow cytometric detection of CD38 on plasma cells, potentially resulting in false-negative findings. Therefore, in patients receiving CD38-based therapy, it is recommended to monitor the CD138^+^CD38^-^ cell population or to employ alternative strategies for plasma cell identification.	Patient-specific clonal sequences are identified and tracked based on V(D)J rearrangements in immunoglobulin genes, such as IGH-VDJH, IGH-DJH, or IGK.
**Impact of sample heterogeneity**	NGF is susceptible to hemodilution or sampling variability.	NGS depends on initial clone identification to detect clonal hierarchical changes and clonal evolution.

NGF: next-generation flow; NGS: next-generation sequencing

Despite these methodological differences, cumulative evidence has demonstrated that both NGF and NGS confer robust and comparable prognostic value.^78,80,81^ In a study of 106 post-transplant patients, NGF and NGS demonstrated strong concordance (R^2^=0.905), and MRD negativity (10^-5^) was consistently associated with superior 3-year PFS (NGF: 91.4% vs. 50.0%; NGS: 88.7% vs. 56.6%) and OS (NGF: 96.6% vs. 74.9%; NGS: 96.2% vs. 77.3%) compared with MRD positivity, highlighting their prognostic significance in MM.^78^ A prospective comparison study showed that achieving MRD negativity (10^-5^) was associated with superior 3-year PFS (NGF: 82.9%; NGS: 84.8%), which further improved with sustained MRD negativity ≥6 months (NGF: 96.7%; NGS: 92.3%), with MRD levels showing strong concordance between NGF and NGS (r=0.9295; P<0.0001).^80^ Similarly, a retrospective study reported a 79.1% concordance between NGF and NGS for MRD monitoring.^81^

While achieving MRD negativity at 10^-5^ already confers marked improvement in outcomes, deeper sensitivity levels (10^-6^) provide incremental prognostic refinement.^8,72,79,82^ In the phase II single-arm study (NCT04113018), 54% of patients achieved ≥CR after induction, with MRD negativity rates of 77% (10^-5^) and 31% (10^-6^) by NGF, and 59% (10^-5^) and 41% (10^-6^) by NGS.^82^ Over time, responses deepened, with ≥CR rising to 66.7% and sustained MRD negativity achieved in up to 87% (10^-5^) and 72% (10^-6^) by NGF, and 77% (10^-5^) and 72% (10^-6^) by NGS of patients at data cutoff. The MRD2STOP study demonstrated that negativity at deeper thresholds (e.g., 10^-6^ or 10^-7^) is consistently associated with superior outcomes, reinforcing the principle of “the deeper, the better”.^72^ A meta-analysis of 44 studies confirmed that MRD negativity strongly correlates with improved PFS and OS, with progressively greater benefit at deeper thresholds (PFS: HR, 0.38 at 10^-4^, 0.31 at 10^-5^, and 0.22 at 10^-6^; OS: HR, 0.50 at 10^-4^, 0.39 at 10^-5^, and 0.26 at 10^-6^; all P<0.001).^8^ However, achieving deeper sensitivity may be limited by technical constraints such as BM sample adequacy or hemodilution; therefore, a minimum sensitivity of 10^-5^ is required to ensure MRD’s broad applicability as a surrogate biomarker. In summary, NGF and NGS differ in their methodology but converge in prognostic power.

#### Level of consensus

82% (14) agree; 18% (3) neutral.

Total: 17 voters.

### Chapter 4: Development of new detection technologies and possible solutions

Do you agree that peripheral residual disease (PRD) detection will become a key direction for future disease monitoring?

#### Statement

PRD detection represents a key direction for future disease monitoring (B1).

#### Discussion

While BM-based MRD testing remains the standard in clinical trials, its invasiveness and impracticality for frequent use in routine practice have prompted growing interest in less invasive PRD assays. The sequential use of MRD and PRD represents a potential next frontier in response assessment for MM, offering several advantages.^13,83–85^

- Minimally invasive and feasible for frequent monitoring
  PRD enables residual disease assessment through peripheral blood, improving patient comfort and allowing longitudinal monitoring without compromising quality of life.^13,83–85^ Detection of circulating tumor DNA, circulating cell-free DNA, MM cell-derived monoclonal immunoglobulin proteins (M-protein), or circulating tumor cells (CTC) offers a practical alternative to repeated BM aspirations, making PRD testing more feasible for long-term management.^13,83–86^
- Biological rationale
  PRD detection accounts for the spatial heterogeneity of MM, where patchy marrow infiltration may be missed by single-site biopsies.^84–86^ Blood-based assays avoid issues such as hemodilution and provide biologically meaningful insights into residual disease.^85^
- Prognostic value
  Accumulating evidence shows PRD to be a robust prognostic biomarker, with PRD negativity linked to the most durable PFS and OS, while persistent PRD indicates higher relapse risk despite clinical remission.^13^ In patients with NDMM not planned for upfront transplant, those with negative PRD at both 3 and 6 cycles post-chemotherapy had superior outcomes (3-year PFS 55.3% vs. 33.3%; median OS not reached vs. 52 months; HR, 3.25; P=0.004), demonstrating that PRD assessment is a robust, noninvasive prognostic biomarker.^85^
- Real-world validation
  In a real-world MM study, CTC detection by NGF emerged as an independent prognostic marker for PFS, with a median PFS of 46 months in MRD-negative patients, an overall HR of 5.1 (95% CI, 2.9–8.9; P<0.0001), and an HR of 7.4 (95% CI, 3.0–18.2; P<0.0001) in patients achieving ≥CR.^83^ BM-based MRD also demonstrated prognostic significance, with an HR of 6.1 (95% CI, 1.5–24.4; P=0.01) in patients achieving ≥CR, highlighting that PRD assessment provides prognostic value comparable to and complementary with BM-based MRD.
- MS-based tracking of PRD
  Quantitative immunoprecipitation-MS (QIP-MS) enables highly sensitive detection of serum M-protein and demonstrated prognostic performance comparable to NGF-based BM-based MRD in GEM2012MENOS65 and GEM2014MAIN.^86^ Concordance between QIP-MS-based PRD and NGF-MRD was high across treatment phases (82% post-induction, 77% post-transplant, 76% post-consolidation, and 85% after 2 years of maintenance), supporting their equivalence as MRD assessment tools. Sustained QIP-MS or NGF positivity was associated with shorter PFS (median 4.04 and 3.9 years, respectively), while conversion from negative to positive by QIP-MS predicted imminent relapse, highlighting its clinical utility.
  A retrospective real-world study of 343 paired MS and NGS assessments from 269 patients with secretory MM showed that matrix-assisted laser desorption ionization time of flight (MALDI-TOF) MS-based tracking of PRD had high specificity, with 95% in NDMM and 84% in RRMM.^87^ It also distinguished therapeutic monoclonal antibodies in 38 cases that might have been misclassified as disease-related. Although concordance with NGS was 65%, MALDI-TOF MS enabled detection and tracking of monoclonal proteins, supporting its potential for residual disease monitoring. A key limitation is that low-level monoclonal proteins without a prior reference above 20 mg/dL are not reported as positive, which may contribute to many discordant cases.
  A retrospective study (NCT05536700) assessed the prognostic value of EasyM, an MS-based assay for PRD detection in MM.^88^ Using receiver operating characteristic curve analysis, an MRD-negative cutoff of <1.86% of diagnostic M-protein levels was defined, at which EasyM demonstrated high sensitivity (95.8% vs. NGF) and specificity (93.2% vs. NGF). In patients achieving CR, EasyM detected persistent or rising M-protein levels and predicted relapse months before conventional assessments. Notably, its prognostic performance was comparable to NGF-based BM-based MRD, while providing the added benefits of earlier detection and noninvasive monitoring, supporting its integration into clinical practice to guide timely treatment adaptation.
- A hybrid approach
  To avoid obtaining multiple BM aspirates at shorter intervals, a hybrid monitoring model is emerging, combining BM-based MRD testing every 24 months with PRD assessment every 6 months using NGF, NGS, or MS.^13^ Sustained PRD negativity may serve as a surrogate for BM-based MRD negativity, while PRD resurgence can indicate relapse without the need for BM aspiration. Moreover, imaging modalities such as positron emission tomography/computed tomography provide complementary value throughout treatment, particularly in patients with extramedullary disease.

Taken together, PRD detection offers minimal invasiveness, biological relevance, strong prognostic power, and real-world applicability. Integrating PRD with BM-based MRD and imaging can provide more precise disease tracking, facilitate adaptive therapy, and reduce patient burden, establishing PRD as a transformative direction for MM monitoring.

#### Level of consensus

82% (14) agree; 18% (3) neutral.

Total: 17 voters.

## Discussion

MRD transcends traditional response assessments by providing a window into the interaction between disease and treatment over time.^4,6,7^ Although it is a strong patient-level surrogate, MRD has limited current use in individualizing treatment.^13^ To address this gap, the consensus panel reviewed the literature and developed expert statements in four areas: treatment goals for CD38-based therapy, MRD-guided decision-making, standardization of MRD detection methods, and integration of novel technologies.

MRD negativity can provide a survival benefit even in high-risk populations.^89,90^ However, its value as a high-risk prognostic marker remains controversial.^13^ Patients with ultra-high-risk disease (≥2 HRCAs) still have a poor prognosis after achieving MRD negativity.^28,91^ Persistent MRD negativity (≥12 to 24 months) may mitigate the negative effect of HRCAs.^92,93^ MRD negativity does not equate to a cure but reflects tumor biology, treatment response, and patient-specific factors.^7,13^ Factors like ISS stage III, detectable CTCs (≥0.01%), multiple HRCAs, or delayed achievement of MRD negativity are associated with MRD resurgence or progression.^94,95^ These highlight the importance of risk-adaptive MRD strategies.

Applying these insights in routine clinical practice remains challenging. Survival outcomes for patients achieving undetectable MRD are generally better in clinical trials than in routine practice.^6,13^ This discrepancy likely reflects differences in patient selection, treatment regimens, and MRD assessment methodologies.^8,93,96^ Clinical trials use standardized therapies and rigorous monitoring, while real-world practice is more heterogeneous, with variable access to novel regimens and less consistent MRD testing. Deeper MRD responses correlate with improved survival, and discrepancies between settings are therefore expected.^11,72^ To ensure consistency and optimize prognostic value, the IMWG consensus criteria for MRD assessment^21^ should be applied in clinical practice.

Although BM-based MRD remains the gold standard for assessing disease sensitivity, interest in PRD detection is increasing as a key direction for future disease monitoring.^4,12^ PRD testing is being explored during maintenance or observation phases; however, discordance between PRD and BM-based MRD may cause false-negative results in 8–21% of patients with persistent BM-based MRD.^86,97,98^ Advances such as more sensitive assays (e.g., CTC analysis by BloodFlow),^98^ multimodal PRD approaches (BloodFlow, CloneSight, and QIP-MS),^99^ and additional circulating biomarkers (such as soluble B-cell maturation antigen)^100^ may reduce this limitation.

The expert panel supports the MRD-adapted strategies to guide clinical decision-making in MM, emphasizing their potential to enhance personalized treatment and improve patient outcomes. Although all 17 panelists are based in the Pan-Pacific region, the recommendations reflect regional clinical practices, treatment access, and healthcare infrastructure. We acknowledge that treatment paradigms, resource availability, and reimbursement policies vary globally; these recommendations should be interpreted within local contexts. Continued international collaboration and validation will be essential to further refine and extend these insights for broader global applicability.

### Authors’ Contribution

**Conceptualization**: Wenming Chen; **Resources**: Wenming Chen, Zhen Cai, Chor Sang Chim, Wee Joo Chng, Juan Du, Chengcheng Fu, Wen Gao, Ichiro Hanamura, Jian Hou, Jeffrey Shang-Yi Huang, Tadao Ishida, Chunrui Li, Aijun Liu, Vadim Ptushkin, Naoki Takezako, Raymond Siu Ming Wong, Dok Hyun Yoon; **Writing – original draft**: Wenming Chen; **Writing – review & editing**: Zhen Cai, Chor Sang Chim, Wee Joo Chng, Juan Du, Chengcheng Fu, Wen Gao, Ichiro Hanamura, Jian Hou, Jeffrey Shang-Yi Huang, Tadao Ishida, Chunrui Li, Aijun Liu, Vadim Ptushkin, Naoki Takezako, Raymond Siu Ming Wong, Dok Hyun Yoon.

### Competing of Interest – COPE

The authors declare that they have no known competing financial interests or personal relationships that could have appeared to influence the work in this paper.

### Ethical Conduct Approval – Helsinki – IACUC

Not applicable.

### Informed Consent Statement

All authors and institutions have confirmed this manuscript for publication.

## Data Availability

Not applicable.
